# Supplementary material for: Redesigning error control in cross-linking mass spectrometry enables more robust and sensitive protein-protein interaction studies
Source: Mol Syst Biol. 2024 Dec 9;21(1):90–106. doi: 10.1038/s44320-024-00079-w (PMC11696718; doi:10.1038/s44320-024-00079-w)
Supplement: Supplementary file 2 — Expanded View Figures [file 44320_2024_79_MOESM2_ESM.pdf]

Expanded View Figures

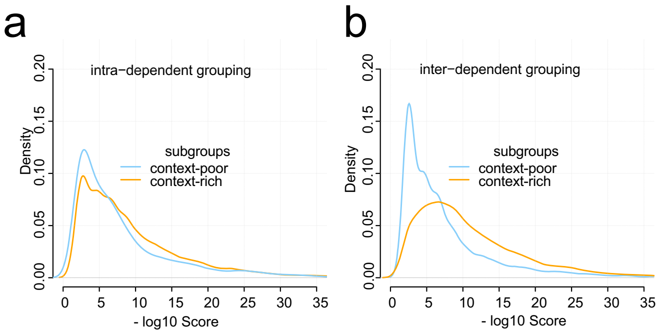

Figure EV1. Divergent error rates in context-dependent inter-link subgroups.

(**A**, **B**) Score distributions of context-rich and context-poor groups upon intra-dependent subgrouping (**A**) or inter-dependent subgrouping (**B**) strategy in the "HEK293T" dataset.

© The Author(s)

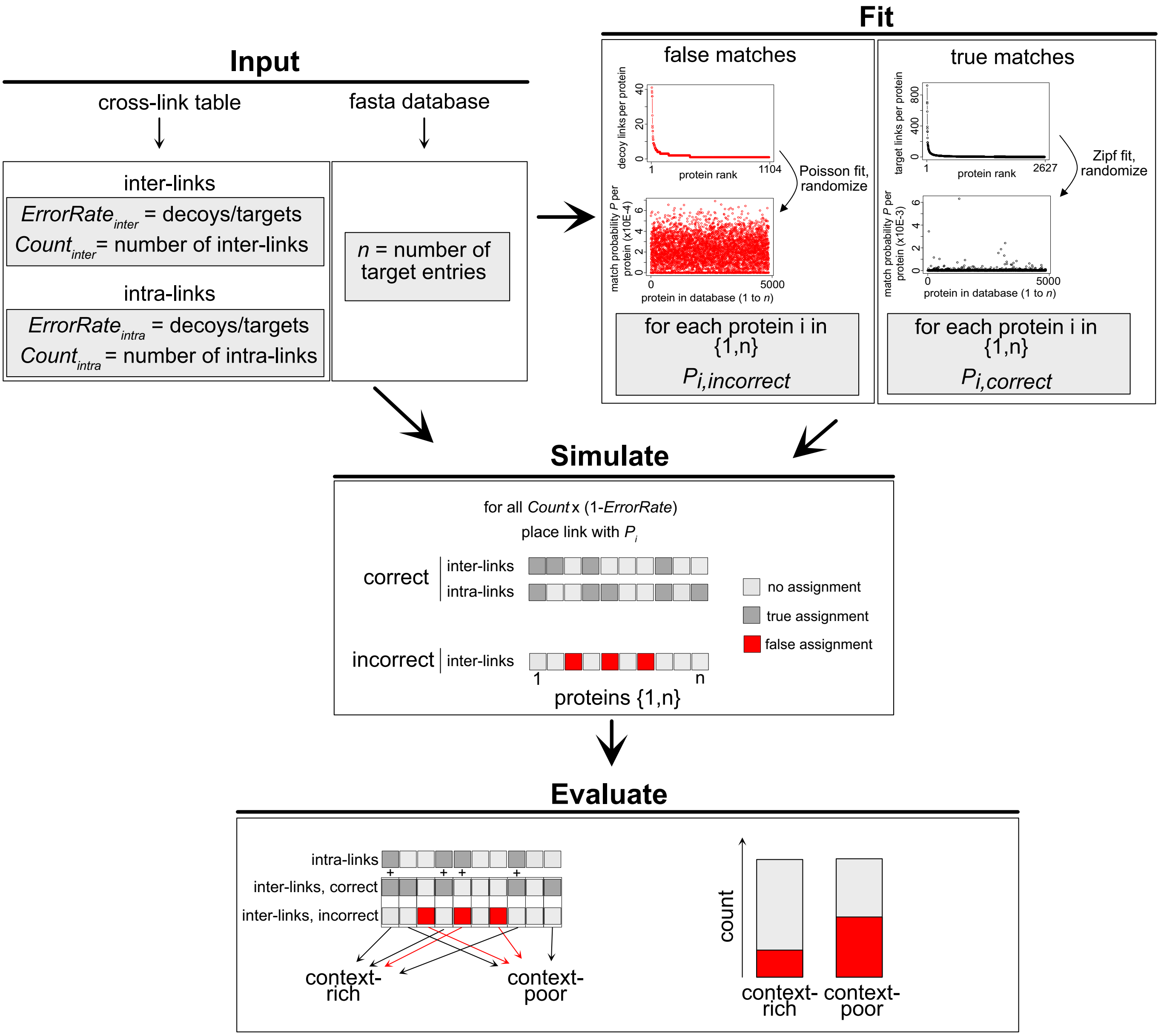

Figure EV2. Simulating false positives in XL-MS datasets.

Workflow for simulations. Database size, the number of cross-links (inter and intra) and their decoy/target fractions were used as input. Protein-dependent probabilities for wrong matches were obtained from a Poisson fit through the decoy cross-link count per protein. Protein-dependent probabilities for correct matches were obtained from a Zipf fit through the target cross-link count per protein (Fit). Correct and Incorrect inter-links were deposited according to these probabilities on the proteins in the database. Incorrect intra-links were not considered. False and true positives were evaluated by grouping them into context-rich and context-poor groups, as schematically depicted for intra-dependent subgrouping.

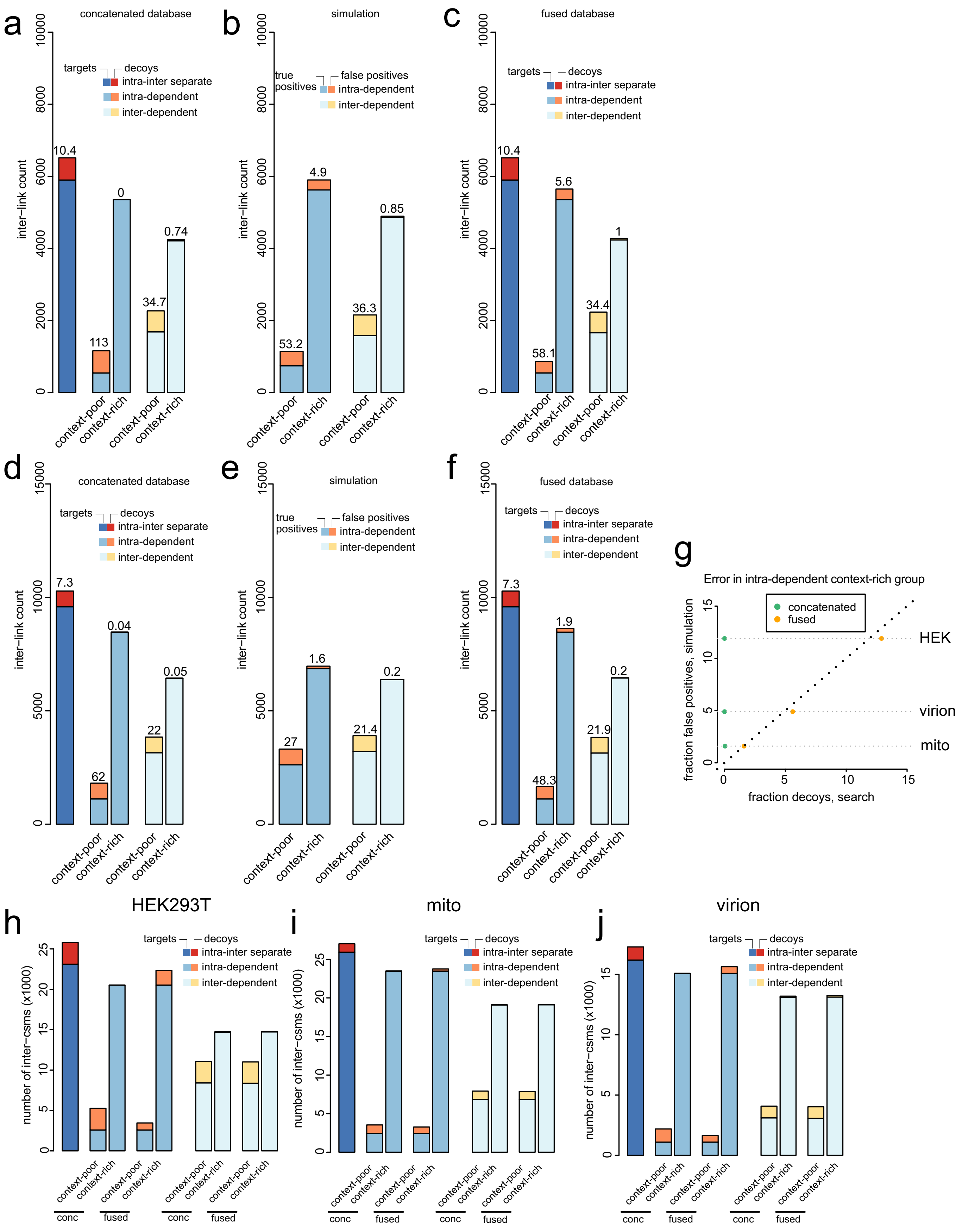
a
concatenated database
targets
decoys
intra-inter separate
intra-dependent
inter-dependent
inter−link count
10.4
113
0
34.7
0.74
context-poor
context-rich
b
simulation
true positives
false positives
53.2
4.9
36.3
0.85
c
fused database
10.4
58.1
5.6
34.4
1
d
7.3
62
0.04
22
0.05
e
27
1.6
21.4
0.2
f
7.3
48.3
1.9
21.9
0.2
g
Error in intra-dependent context-rich group
concatenated
fused
HEK
virion
mito
fraction false positives, simulation
fraction decoys, search
h
HEK293T
number of inter−csms (x1000)
conc
fused
i
mito
j
virion

 © The Author(s)

Figure EV3. Target-decoy fusion agrees with simulated error rates upon context-sensitive subgrouping.

(**A–F**) Distribution of inter-link matches to target or decoy entries in the "virion" (**A**, **C**) or "mito" (**D**, **F**) dataset upon intra-inter separate grouping or subgrouping of inter-link matches according to intra- and inter-dependent criteria using a concatenated (**A**, **D**) or fused (**C**, **F**) database. Simulation data on the distribution of false and true positives in context-rich and context-poor subgroups is given in panels (**B**) for "virion" and (**E**) for "mito" datasets. The number on top of the bars corresponds to the fraction of decoys to targets (**A**, **C**, **D**, **F**) or false positives to true positives (**B**, **E**) in percent. (**G**) Inter-link FDR in the context-rich, intra-dependent subgroup upon simulation, and upon fused or concatenated search for three biological datasets. The diagonal dotted line indicates a full agreement between simulation and search. (**H–J**) Distribution of inter-CSMs in different subgroups, as indicated for HEK293T, mito and virion datasets.

© The Author(s)

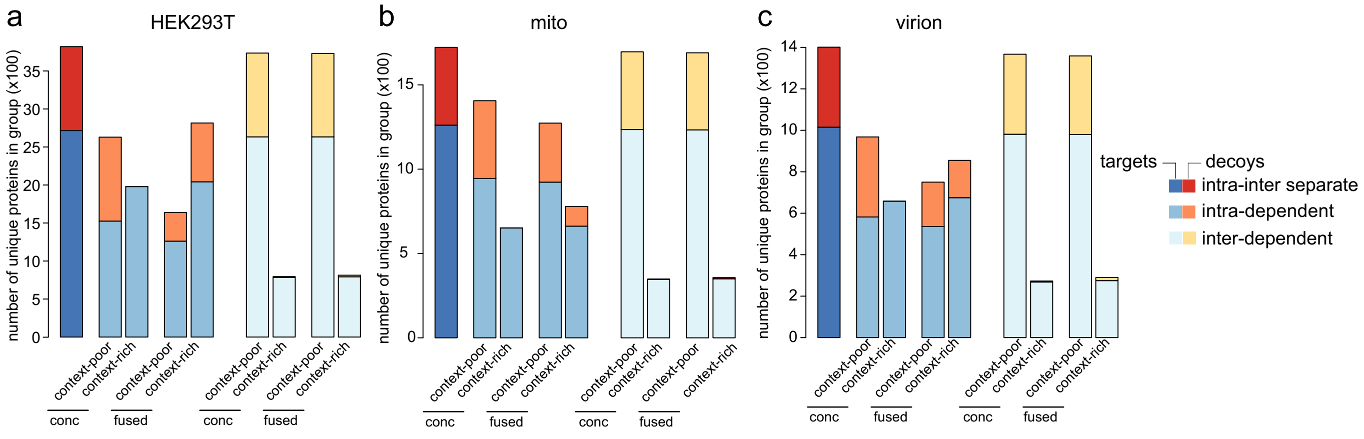

Figure EV4. Unique Proteins in different subgroups.

(**A–C**) Unique target or decoy proteins in subgroups from the HEK293T (**A**), mito (**B**) or virion (**C**) dataset upon intra-inter separate grouping or subgrouping of inter-link matches according to intra- and inter-dependent criteria using a concatenated or fused databases.

 © The Author(s)

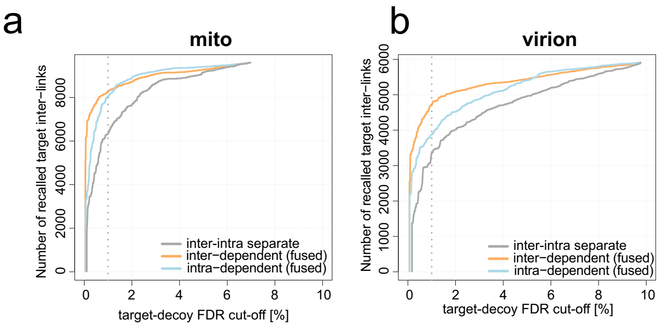

Figure EV5. Improvement in statistical power through context-sensitive subgrouping.

Recall of target inter-links as a function of the FDR cutoff comparing different data grouping strategies on a fused database for the (**A**) mito and (**B**) virion datasets.

© The Author(s)
